# Different acupotomy for stenosing tenosynovitis: A protocol for systematic review and network meta-analysis

**DOI:** 10.1097/MD.0000000000028050

**Published:** 2022-01-07

**Authors:** Lingzhi Wei, Qi Tong, Yue Liu, Xinju Hou, Fang Zhi

**Affiliations:** a Nanchang Hongdu Hospital of Traditional Chinese Medicine, Jiangxi Province, China; b Jiangxi Health Vocational College, Jiangxi Province, China.

**Keywords:** needle knife, network meta-analysis, protocol, stenosing tenosynovitis

## Abstract

**Background:**

Stenosing tenosynovitis (STS) is a chronic aseptic inflammation caused by mechanical friction. The main clinical manifestations are local pain and limited activity of the affected parts, which reduce people's quality of life. The clinical effect of acupotomy in the treatment of STS is significant, and the operation is simple and the side effect is small. But there are many kinds of acupotomology, and there is a lack of comparative study between different Acupotomology. In this study, the effectiveness of 4 commonly used needle knife therapies (v-knife, oblique knife, crochet knife, flat knife) was ranked by the method of network meta.

**Methods:**

CNKI, Wanfang, VIP, Sinomed, PubMed, and Cochrane Library were searched to collect randomized controlled trials of v-knife, oblique knife, crochet knife, and flat knife in the treatment of STS. The search time limit is from the date of establishment to October 15, 2021. Revman5.3, gemtc 0.14.3, and stata14.2 were used for data analysis, and Cochrane bias risk assessment tool was used to screen and evaluate the quality of included literatures.

**Conclusion:**

Objective to provide evidence-based medicine evidence for clinical selection of the best needle knife treatment scheme for STS.

## 1 Introduction

Stenosing tenosynovitis (STS) is a chronic aseptic inflammation caused by mechanical friction, including STS of flexor digitorum tendon and STS of radial tendon.^[1]^ The main clinical manifestations are local pain and activity limitation of the affected parts, which affect people's activities of daily living and reduce people's quality of life.^[2]^ The incidence rate of mobile phone is more common in handicraft workers and housewives. But with the rapid development of modern electronic products such as cell phones and computers, the incidence rate is increasing. At present, Western medicine commonly used closure, surgery, and other treatment methods, because of the side effects of closure and invasive surgical treatment, so in clinical use is limited.^[3–6]^ Acupotomy, as a characteristic therapy of traditional Chinese medicine, is widely used in the clinic because of its simple operation, small side effect, and good clinical effect.

At present, there are many clinical reports on the treatment of STS with acupotomy, and the clinical effect is more significant.^[7–9]^ However, there are many kinds of acupotomy, and the treatment advantages are not the same. There is a lack of comparative study between different acupotomy treatments, which brings trouble to the choice of clinicians. In order to provide evidence-based medical evidence for the clinical selection of the best needle knife treatment for STS, the effectiveness of 4 commonly used needle knife therapies (v-knife, oblique knife, crochet knife, and flat knife) was ranked by the method of mesh meta.

## 2 Protocol registration

This system review program will strictly follow the system review and meta-analysis program (PRISMA-P) preferred report items for reporting.^[10]^ The system review program has been registered on the INPLASY website (the registration number is INPLASY202150059). If there are any adjustments during the entire study period, we will fix and update the detailed information in the final report in time.

## 3 Methods

### 3.1 Inclusion and exclusion criteria

#### 3.1.1 Study type

Randomized controlled trials (RCTs) based on different needle scalpels for STS were conducted in Chinese and English only. The exclusion criteria were as follows:

1. Non-RCTs, such as systematic reviews, reviews, animal experiments, etc.
2. Using nonstandard grouping methods, such as coin tossing, odd and even numbers, or patients willing to group, etc.
3. There is no definite diagnostic or therapeutic criteria.
4. The experimental group and the control group contained other interference therapy.
5. The latest one was selected for the repeated articles published in Chinese and English journals.
6. Data or full-text literature cannot be obtained.

#### 3.1.2 Participants

The diagnosis of STS has clear and accepted diagnostic criteria and therapeutic criteria; there are no restrictions on age, race, gender, and source of cases. The following patients were excluded:

1. Cannot stand acupotomy or patients with low compliance;
2. Patients with severe organic diseases;
3. Pregnant women;
4. Psychiatric patients may not be able to accurately describe the symptoms caused by unconsciousness.

#### 3.1.3 Interventions

The treatment group was only treated with acupotomy (including v-knife, oblique knife, crochet knife, and flat knife), while the control group was treated with closed therapy, conventional surgery, and flat knife (both the treatment group and the control group could cooperate with the treatment of basic diseases of internal medicine).

#### 3.1.4 Outcome indicators

The included outcome indicators included 1 or more of the following: cure rate, total effective rate (effective rate = ([cured + markedly effective + effective]/total number of cases × 100%), complications, recurrence rate.^[11–13]^

### 3.2 Data sources and search strategies

**Figure 1:**
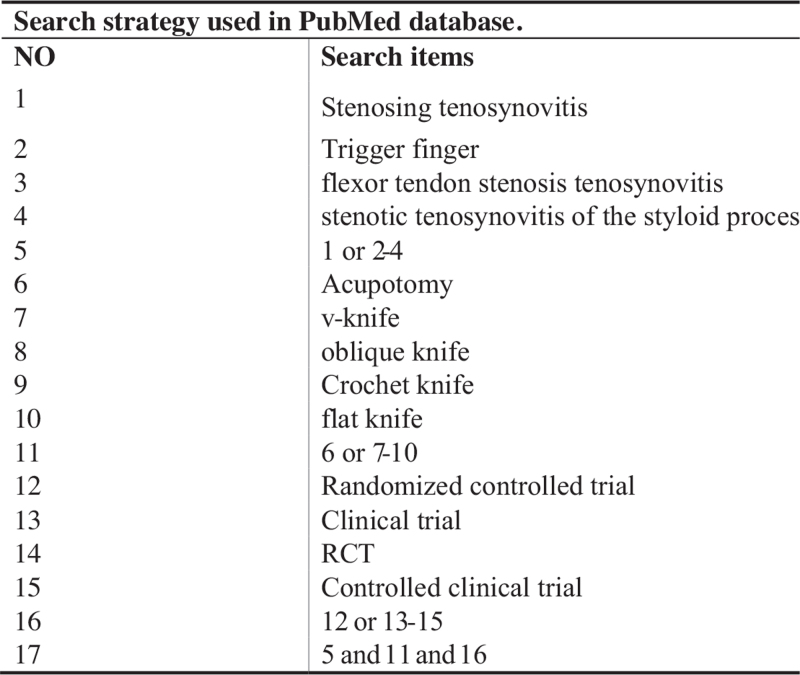
Search strategy used in PubMed database.

RCTs of needle knife in the treatment of STS were searched in CNKI, Wan-Fang data, CBM, VIP, PubMed, and Cochrane Library. The keywords were “Acupotomy” or “v-knife” or “oblique knife” or “Crochet knife” or “flat knife” and “Stenosing tenosynovitis” or “trigger finger” or “flexor tendon stenosis tenosynovitis” or “stenotic tenosynovitis of the styloid process,” and the literature retrieval time was from the database establishment to October 15, 2021. The retrieval strategy is to combine keywords with subject words and free words. The data retrieval strategy is shown in Figure 1.

### 3.3 Selection of studies and data extraction

Two reviewers read the title, abstract and full text of the literature extracted the literature and literature research data that met the inclusion criteria for independent screening and exclusion criteria and then cross-checked. In case of any objection, it will be decided by the third-party assessor. This is to create an information extraction table in Excel. The extracted information includes: author, publication time, number of cases, allocation method, intervention measures, course of treatment, and outcome indicators.

### 3.4 Risk assessment of bias

Two reviewers used the Cochrane Handbook of systematic review to evaluate the quality of the included articles and assess the risk of bias, including selection bias, implementation bias, measurement bias, follow-up bias, reporting bias and other source bias. The results of the evaluation are “high,” “risk,” “low risk,” and “unclear risk.”^[14,15]^

### 3.5 Statistical analysis

Revman 5.3 software was used to assess the risk of bias. Stata 14.2 and gemtc 0.14.3 software were used to conduct network meta-analysis using Markov chain Monte Carlo (MCMC).^[16,17]^ Stata was used to calculate the SUCRA (surface under the cumulative ranking curves, SUCRA) value and area under the curve, so as to rank the efficacy of various interventions. When there is a closed loop in the network diagram, the inconsistency test is needed. If there is no obvious inconsistency between the 2, Bayesian inference is performed in the consistency model, and the convergence of the results is evaluated by the potential scale reduced factor (potential scale reduction factor, PSRF). When 1.00 ≤ PSRF ≤ 1.05, the convergence of the results is good.^[18]^

### 3.6 Assessment of inconsistency

This study involves many interventions. The inconsistency between direct evidence and indirect evidence should be evaluated by Stata software. The 95% confidence interval starts from 0.^[19]^ If *P* > .05, it indicates that there is no obvious inconsistency between the direct and indirect comparison of each intervention measure, indicating that the consistency is good. At the same time, the node segmentation model is used to determine each node.^[20]^

### 3.7 Sensitivity analysis

The purpose of sensitivity analysis is to eliminate low-quality noise research and explore the source of heterogeneity. Then, the reliability and stability of the results were analyzed by observing the heterogeneity of different studies and whether the results changed after treatment.

### 3.8 Assessment of publication bias

Funnel plot if outcome measures included in the study are ≥10 it will be used to evaluate the publication bias of included trials.^[21]^ If there are differences in symmetry or distribution, it indicates bias or small sample effect.

### 3.9 Ethics and dissemination

Because this is a systematic review of the protocol and a network meta-analysis, all the data in this study are from published studies and do not involve patients, so there is no need for ethical recognition. The results of this study will be distributed to peer reviews and presented at relevant meetings.

## 4 Discussion

STS is a common chronic sports injury disease, which can lead to joint pain, flexion and extension disorders, and affect people's daily life. Current studies have shown that acupotomy through minimally invasive release can well relieve patients’ acute pain, improve joint activity, and improve the quality of life of patients. With the wide application of acupotomy in clinic, many relevant meta-analyses have proved the advantages of acupotomy in the treatment of STS.^[9]^ However, the traditional meta-analysis can only compare 2 kinds of intervention measures, usually simple needle knife compared with closed therapy. At present, there are many improved needle knife therapies in China, with higher clinical effectiveness and safety. However, there is a lack of comparison between different knives, so it is difficult to provide guidance for clinicians. Network meta-analysis can provide the best treatment plan for clinic by quantifying the treatment effect of various needle knife therapies.

## Author contributions

**Conceptualization:** Lingzhi Wei.

**Methodology:** Lingzhi Wei, Qi Tong, Xinju Hou.

**Project administration:** Yue Liu.

**Software:** Xinju Hou.

**Supervision:** Fang Zhi.

**Writing – original draft:** Lingzhi Wei.

**Writing – review & editing:** Lingzhi Wei, Fang Zhi.
